# Statistics of Medical Schools, Sessions 1848-9

**Published:** 1849

**Authors:** 


### ARTICLE III.

### STATISTICS OF MEDICAL SCHOOLS, SESSIONS 1848-9.

We have collected from various sources all the reliable information at our command upon the subject into the following table. Of many of the schools we have no positive information, but believe the agregate will show quite a falling off from the preceding Sessions in the number of Students.

| Colleges. | No. of Students. | Graduates. |
|---|---|---|
| College of Physicians and Surg. N. York. | 176 | 39 |
| University of Maryland. | 190 | 63 |
| Albany Medical College. | 101 | |
| Medical College of Ohio. | 175 | |
| Transylvania University, Lexington Ky. | 120 | 49 |
| Mass. Med. Col. Boston. | | 25 |
| Starling Med. College. | 173 | 50 |
| University of the City of N. Y. | | 134 |
| Geneva Med. College. | | |
| Rush Med. College. | 100 | 20 |
| Med. Col. of Georgia. | 133 | 36 |
| Jefferson Med. Col. Phila. | | 188 |
| Cleveland Med. Col. | 251 | |
| University of Louisville. | 331 | 81 |
| University of the State of Missouri. | 154 | |

